# Supplementary material for: Nepali oral microbiomes reflect a gradient of lifestyles from traditional to industrialized
Source: bioRxiv. 2024 Jul 3:2024.07.01.601557. Preprint. [Version 1] doi: 10.1101/2024.07.01.601557 (PMC11244963; doi:10.1101/2024.07.01.601557)
Supplement: Supplement 10 [file NIHPP2024.07.01.601557v1-supplement-10.pdf]

bioRxiv preprint doi: https://doi.org/10.1101/2024.07.01.601557; this version posted July 3, 2024. The copyright holder for this preprint (which was not certified by peer review) is the author/funder, who has granted bioRxiv a license to display the preprint in perpetuity. It is made available under aCC-BY 4.0 International license.

Additional Files

Additional file 1 - Supplementary Figures.docx

- Title of data: Supplementary Figures
- Description of data: **S1 Figure**: Confusion matrix of random forest classification based on survey data. **S2 Figure**: Correspondence analysis based on survey data. **S3 Figure**: Microbiome composition within sample is similar across the two extraction kits tested.

bioRxiv preprint doi: https://doi.org/10.1101/2024.07.01.601557; this version posted July 3, 2024. The copyright holder for this preprint (which was not certified by peer review) is the author/funder, who has granted bioRxiv a license to display the preprint in perpetuity. It is made available under aCC-BY 4.0 International license.

S4 Figure: All alpha diversity metrics across lifestyle groups with DNA extracted using the Qiagen kit. **S5 Figure**: Alpha diversity metrics with DNA extracted using the PowerSoil kit. **S6 Figure**: Oral microbiome composition extended figures - Axis 2 from Qiagen kit extraction, and ordinations on samples extracted using the PowerSoil kit. **S7 Figure**: Oral microbiome composition calculated with unweighted Unifrac distance. **S8 Figure**: Oral microbiome composition calculated with weighted Unifrac distance. **S9 Figure**: Confusion matrix of Random Forest classification based on microbiome data. **S10 Figure**: Correlations between diversity metrics and CA axes. **S11 Figure**: Smoking and *sisnu* are associated with several differentially abundant taxa. **S12 Figure**: Predicted functional abundance significantly differs by lifestyle. **S13 Figure**: Metabolism pathways form the majority of the significantly differentially abundant predicted functions from PICRUSt2. **S14 Figure**: Microbiome functional enrichment analysis. **S15 Figure**: *Fusobacterium* contributes to predicted platinum resistance, which varies by lifestyle. **S16 Figure**: Correlations between the top three oral and gut microbiome PCoA axes. **S17 Figure**: Rarefaction curves. **S18 Figure**: Read depth across samples after read QC. **S19 Figure**: Comparison of antibiotic use on oral microbiome diversity. **S20 Figure**: Final sample size per lifestyle per extraction kit. **S21 Figure**: Read depth against lifestyle and *Brachymonas* relative abundance.

Additional file 2 - S1 Table.xlsx

- Title of data: **S1 Table - Sequence, survey, population, and questionnaire info of the sampled individuals**
- Description of data: Tab 1 describes survey and sequence metadata data collected. Column abbreviations and responses are explained in greater detail in Tab 3. Column names that end with “2” contain responses that were categorized and transformed to a scale of 0-3, in which possible values for binary variables are 0 or 3 (ie. sex) and possible values for continuous variables are 0, 1, 2, or 3 (ie. fuel source). No survey data was collected for the American Industrialists. Tab 2 describes the lifestyle pertaining to each population and their sample sizes. Tab 3 contains the survey questionnaire, including the codes pertaining to each question asked and list of possible responses.

Additional file 3 - S2 Table.csv

bioRxiv preprint doi: https://doi.org/10.1101/2024.07.01.601557; this version posted July 3, 2024. The copyright holder for this preprint (which was not certified by peer review) is the author/funder, who has granted bioRxiv a license to display the preprint in perpetuity. It is made available under aCC-BY 4.0 International license.

- Title of data: **S2 Table - Oral microbiome differential abundance results from ALDEx2**
- Description of data: Oral microbiome genera tested for differential abundance across lifestyle. Overall, 2/111 genera were identified as significantly differentially abundant. Kruskal-Wallis module was utilized and p-value correction was applied using the Benjamini-Hochberg method. Both unadjusted (kw.ep) and adjusted p-values (kw.eBH) are shown, and adjusted p-value < 0.05 is the threshold for significance.

Additional file 4 - S3 Table.csv

- Title of data: **S3 Table - Results of genera tested for following the lifestyle gradient.**
- Description of data: All genera tested for following the lifestyle gradient using the Jonckheere-Terpstra test followed by the Benjamini-Hochberg method to correct for multiple tests (BHadj_p_value). Adjusted p-value < 0.05 is the threshold for significance. Nine genera significantly follow the lifestyle gradient.

Additional file 5 - S4 Table.csv

- Title of data: **S4 Table - Associations between differentially abundant microbes and lifestyle factors**
- Description of data: Associations between differentially abundant microbes from the Jonckheere-Terpstra test and lifestyle factors were tested via linear models. Linear models were generated between each microbe and each lifestyle factor and then tested for significance, for a total of 333 tested associations. P-value correction was applied using the Benjamini-Hochberg method. Both unadjusted and adjusted p-values are shown. Adjusted p-value < 0.05 is the threshold for significance.

Additional file 6 - S5 Table.csv

- Title of data: **S5 Table - Predicted functional potential differential abundance results**
- Description of data: PICRUSt2 predicted functions were analyzed for differential abundance based on lifestyle. None of the 107 tested functions were found to be significant after multiple test correction, but 21/107 pathways were significant prior to correction. Kruskal-Wallis module in ALDEx2 was utilized and p-value correction was applied using the Benjamini-Hochberg method. Both unadjusted (kw.ep) and adjusted p-values (kw.eBH) are shown. Adjusted p-value < 0.05 is the threshold for significance.

Additional file 7 - S6 Table.csv

bioRxiv preprint doi: https://doi.org/10.1101/2024.07.01.601557; this version posted July 3, 2024. The copyright holder for this preprint (which was not certified by peer review) is the author/funder, who has granted bioRxiv a license to display the preprint in perpetuity. It is made available under aCC-BY 4.0 International license.

- Title of data: **S6 Table - Samples overlapping between the gut and oral microbiome studies**
- Description of data: List of samples overlapping between the gut and oral microbiome studies, along with the samples unique to each microbiome study. The first column "both" lists sample IDs that are associated with both gut and oral samples. The second column "gut_only" lists sample IDs that are associated with only gut samples. The third column "oral_only" lists sample IDs that are associated with only oral samples.

Additional file 8 - S7 Table.csv

- Title of data: **S7 Table - Gut microbiome differential abundance results from ALDEx2**
- Description of data: Gut microbiome genera analyzed for differential abundance based on lifestyle via ALDEx2. Overall, 37/136 genera were identified as significantly differentially abundant. Kruskal-Wallis module was utilized and p-value correction was applied using the Benjamini-Hochberg method. Both unadjusted (kw.ep) and adjusted p-values (kw.eBH) are shown. Adjusted p-value < 0.05 is the threshold for significance.

Additional file 9 - S8 Table.csv

- Title of data: **S8 Table - Read counts through each sequence processing step**
- Description of data: Read counts at each step of sequence processing, starting with raw demultiplexed reads and all the way through DADA2, merging, and chimera removal. "Input" column refers to the number of raw reads obtained per sample after sequencing, "filtered" refers to the number of reads remaining after initial read QC, "denoised" refers to the number of reads remaining after denoising in DADA2, "nochim" refers to the number of reads remaining after chimeric sequences were removed, and "retained_overall" is the total proportion of reads retained following all QC steps from the input amount. Table does not include the samples that failed to pass initial read QC.

Additional file 10 – Nepali abstract.pdf

- Title: Nepali Language Abstract
- Description: Summary of study findings in Nepali language as translated by Aashish R. Jha
